# Water, energy and climate benefits of urban greening throughout Europe under different climatic scenarios

**DOI:** 10.1038/s41598-021-88141-7

**Published:** 2021-06-09

**Authors:** Emanuele Quaranta, Chiara Dorati, Alberto Pistocchi

**Affiliations:** 1 European Commission, Joint Research Centre (JRC), Ispra, Italy; 2 ARHS, Ispra, Italy

**Keywords:** Ecology, Climate sciences, Ecology, Environmental sciences, Environmental social sciences, Hydrology, Energy science and technology, Engineering

## Abstract

Urban greening is an effective mitigation option for climate change in urban areas. In this contribution, a European Union (EU)-wide assessment is presented to quantify the benefits of urban greening in terms of availability of green water, reduction of cooling costs and CO_2_ sequestration from the atmosphere, for different climatic scenarios. Results show that greening of 35% of the EU’s urban surface (i.e. more than 26,000 km^2^) would avoid up to 55.8 Mtons year^−1^ CO_2_ equivalent of greenhouse gas emissions, reducing energy demand for the cooling of buildings in summer by up to 92 TWh per year, with a net present value (NPV) of more than 364 billion Euro. It would also transpire about 10 km^3^ year^−1^ of rain water, turning into “green” water about 17.5% of the “blue” water that is now urban runoff, helping reduce pollution of the receiving water bodies and urban flooding. The greening of urban surfaces would decrease their summer temperature by 2.5–6 °C, with a mitigation of the urban heat island effect estimated to have a NPV of 221 billion Euro over a period of 40 years. The monetized benefits cover less than half of the estimated costs of greening, having a NPV of 1323 billion Euro on the same period. Net of the monetized benefits, the cost of greening 26,000 km^2^ of urban surfaces in Europe is estimated around 60 Euro year^−1^ per European urban resident. The additional benefits of urban greening related to biodiversity, water quality, health, wellbeing and other aspects, although not monetized in this study, might be worth such extra cost. When this is the case, urban greening represents a multifunctional, no-regret, cost-effective solution.

## Introduction

Climate change and the current trends in urbanization make city resilience a clear priority^1^. Urban areas suffer from heat waves^2–4^ and generally require a high amount of energy for the cooling of buildings. Impervious surfaces exacerbate floods and their impacts, because urban runoff is quickly discharged to the receiving water bodies where it may cause disturbance to aquatic ecosystems^5^, and is often a significant source of pollution^6^.

Health and environmental risks due to climate effects in urban areas are expected to increase, especially in developing cities that are experiencing rapid population growth^3,7^. A recent study found that the accumulated total costs resulting from the impact of global and local climate change on cities since the year 2000 were about 2.6 times the costs without urban weather-related effects^7^.

Urban climate has been acknowledged to be strongly modified by human influences^8^. Green areas inside a city can lower the ambient air temperature and adjust the humidity of surrounding areas^9–12^, besides regulating runoff and enabling rainwater harvesting^13,14^, as unsealed soil allows retaining rainwater subsequently available for vegetation to grow.

In the last years, urban greening has attracted considerable interest as a broad-scoped management measure^9^. In the context of urban greening, a frequent option is not to restore unsealed soil, but to cover an impervious surface with a vegetated soil layer, usually on top of a waterproof membrane and a drainage layer to protect the underlying impervious surface. The latter is sometimes the roof of a building (which would be turned into a “green roof”), but could equally be a paved external surface with underlying pipelines or other services. If not specified otherwise , in the remainder of this work we refer to “greened surface” , or equivalently "green roof", as any soil cover of an urban impervious surface, enabling water infiltration and vegetation growth.

While green roofs have been used for centuries, they are now reviving under the current climate and urbanization trends^15^. Essentially, they harvest “blue” rainwater and make it available for evapotranspiration (turning it into “green water”, i.e. water used by vegetation and soil), modifying the hydrologic and energy balance of the surfaces. In addition, rainwater in excess of evapotranspiration may be collected through drainage for possible reuse. Green roofs bring several potential benefits including reduction of storm water runoff by retaining precipitation^13,14,16,17^, reduction of energy demand for the cooling of buildings^18,19^, mitigation of the urban microclimate^19,20^. Moreover, by supporting vegetation growth, they enhance sequestration of carbon dioxide and pollutants from the atmosphere^21,22^, reduce noise in buildings^23^ , provide usable spaces for social activities and horticulture^24–26^ and for wildlife habitat, especially birds and pollinators^27,28^. Because of these multiple benefits, green roofs can be an important urban management measure, meeting the aspirations of the European green deal on buildings renovation^29^.

Having in mind the local benefits of urban greening (single building- or city-scale), in this contribution we present a European-scale quantification of the potential benefits of green roofs in terms of water and climate regulation, energy saving and biomass production (hence carbon sequestration), using the meta-models described in Quaranta et al.^30^. On this basis, we discuss the opportunities and limitations of greening as a tool for sustainable urban development in Europe. The objective of the present paper is not a detailed assessment at single sites but the screening of a comprehensive strategy to develop urban greening as a mainstream solution.

## Materials and methods

Our analysis combines a GIS implementation of the meta-models proposed in^30^ applied to the EU context to quantify the benefits of converting 1 m^2^ of impervious surface into a green surface, with an empirical quantification of costs and benefits. The Net Present Value (NPV) of the investment was also calculated. NPV is an economic valuation analysis that takes into account the difference between the present value of benefits and the present value of costs over a period of time, that in our case was assumed to be 40 years. NPV allows to estimate the profitability of an investment or a project. Therefore, NPV accounts for the time value of money and can be used to compare similar investment alternatives. The NPV relies on a discount rate, that is the rate of return used to discount future cash flows back to their present value.

Quaranta et al.^30^ combined the hydrological model of Pistocchi et al.^31^, with the energy and biomass model of Neitsch et al.^32^, to simulate biomass growth, the water and surface energy balance for 671 functional urban areas (FUA) across Europe at daily time step, using European scale gridded weather time series for the period 1990–2013^33^ as input. The results were used to derive simple meta-models predicting the following indicators as a function of climatic descriptors, i.e. annual precipitation (*P*), annual potential evapotranspiration (*ET0*) and annual actual evapotranspiration (*AET*):

1. the average difference in surface (skin) temperature in summer, Δ*T*_*s*_ (°C), between an impervious urban surface and a greened surface at the same location (Eq. 1);
2. the average difference in summer temperature, Δ*T* (°C), between an impervious urban surface and the bottom of the soil layer, placed for the greening on the urban surface at the same location (Eq. 2);
3. the difference between annual rainfall and annual runoff, *RR* (mm year^−1^), for a greened surface, representing the runoff avoided as a consequence of greening (Eq. 3);
4. the annual biomass that may grow on a greened surface *CB* (kg m^−2^ year^−1^), Eq. (4):
  1 $$\Delta T_{s} = 0.00{61}AET + {1}.{46}$$
  2 $$\Delta T = {6}.{\text{85 ln}}\left( {ET0} \right){-}{27}.{83}$$
  3 $$RR/P = {17}.{8}P^{{ - 0.{544}}}$$
  4 $$CB = {1}.{\text{65 ln}}\left( {ET0} \right) - {8}.{685}$$

In the derivation of the metamodels of Quaranta et al.^30^ *ET0* was computed at daily step with the Penman–Monteith equation by Bisselink et al.^33^, and aggregated as a yearly value. While *AET* is usually estimated with a hydrological model and may not be as readily available as *P* or *ET0*, in the European context it can be very well approximated by a simple Budyko model ^30^ and is therefore considered a climatic predictor on a par with *P* and *ET0* for the purposes of this analysis. The meta-models proved to surrogate the results obtained by solving the integrated hydrological-energy-biomass model with an error usually below 10% quite evenly across the European region^30^.

The above equations are valid for the European context, and for a soil layer thickness of 30 cm covered with an annual herbaceous cover (the meta-models were derived for a generic thickness *t* and proved to be relatively insensitive to the selected herbaceous crop^30^). In the present study we refer to a 30 cm thick soil with the aim of determining the maximum benefits of greening implementation. Higher thickness would imply higher costs, while benefits would not change substantially. A soil of 30 cm may be unfeasible as a uniform cover of roofs in many buildings due to architectural and structural constraints, but could be a reasonable solution when greening e.g. paved ground or subterranean parking lots. In the greening of roofs, patches of 30 cm-thick soil cover on less than 100% of the surface could still be feasible. 

In the analysis presented here, the above indicators (Eqs. 1–4) were computed using the climatic predictors *ET0*, *P* and *AET* for present conditions (1990–2013) and for 2 climatic scenarios represented by regional concentration pathways (RCP) 8.5 and 4.5^34^ for the period 2070–2100, using 4 regional climate models from the Euro-Cordex ensemble ^63^. Therefore, we considered a total of eight climatic scenarios in our estimations. The models included were from the Danish Meteorological Institute (model code used here: DM), Swedish Meteorological and Hydrological Institute (SM), Royal Dutch Meteorological Institute (KN) and Institute Pierre-Simon Laplace (IP). In the following, the scenarios are defined by the above model codes followed by codes 45 or 85 for the two RCPs considered, respectively. *P* and *ET0* needed to compute *AET* under climate scenario conditions were average values for the climate simulation period 2070-2100 for each model. Under each scenario, *P* and *ET0* were computed as the annual average from daily values over the period considered.

The four indicators of Eqs. (1–4) were quantified at the nodes of the regular grid of 5 × 5 km at which the climate variables were available^33^ . The impervious surface (roofs and other surfaces) within each of the 5 × 5 km grid cells was also quantified, so that cumulative curves could be calculated for each indicator, quantifying the area in km^2^ where a certain indicator value was exceeded. The impervious surface area was estimated as per^35^.

The summer temperature difference of Eq. (2), Δ*T,* can be interpreted as the cooling reduction of a roof if covered by soil, which implies a reduced energy demand for cooling. The corresponding energy cost saving *G* was estimated as:

5 $$G = U A\Delta T h \frac{1}{{\text{SEER}}}C$$

where *A* is the area of the roofs, *h* is the amount of hours during summer months (from June to August included), *SEER* (set to 3.1) is the seasonal energy efficiency ratio for Europe^36^, *C* is the electricity cost set to 0.2 Є kWh^−1^^37^ representative of an average value in Europe and *U* is the average transmittance of the roof (set to 0.30 W m^−2^ K^−1^ as a European reference value, Eurima^38^). The carbon emissions are assumed to be 0.325 kg CO_2_ equivalent kWh^−1^, a value corresponding to the European electricity generation ^39^. Obviously, this calculation should be applied only to the impervious surfaces that are represented by roofs of buildings. The impervious surface covered by building roofs was assumed to be 26,450 km^2^ (35% of the total impervious area) as in Bódis et al.^40^. The remaining 65% is represented by urban areas like streets and impervious open spaces assumed to not be amenable to greening.

The carbon sequestered by an annual herbaceous biomass was estimated as 0.35 kg C m^−2^ year^−1^ in Saliendra et al.^41^, and 0.27 kg C m^−2^ year^−1^ in Gilmanov et al.^42^. In this analysis, we considered the more conservative value of Gilmanov et al.^42 ^ that corresponds to 0.98 kg CO_2_ m^−2^ year^−1^. The present carbon dioxide (CO_2_) market price is 22.5 Є tons^−1^ of CO_2_^43^. This amount of carbon is effectively sequestered if the biomass is preserved as straw or if it avoids an equivalent amount of biomass to be mineralized elsewhere, and is therefore an upper limit.

 The residual runoff (i.e. *P*-*RR*) generated by the green surfaces can be in principle harvested, instead of discharging it into the environment, if we provide a sufficient storage volume to buffer demand and availability. In this assessment, we compute the storage volume required to harvest all the runoff generated by a green surface, assuming a constant demand whose yearly cumulate equals the yearly cumulate of available runoff.  The required storage volume was calculated for each FUA using the classic mass diagram analysis^64^ for every year of the time series. The calculations were based on the daily runoff predicted under current conditions with the model described in Quaranta et al.^30^. In particular, we computed the average volume among those required in the various years, *V*_*avg*_, the maximum, *V* _*max*_, and the minimum, *V*_*min*_. We derived an ordinary least squares multiple linear regression model to predict *V*_*avg*_, *V*_*min*_ and *V*_*max*_ (mm)  as a function of the climatic predictors already used for the above indicators.  After testing various combinations of the predictors, we chose the best performing models (with mean absolute error (MAE) of 19% when considering *V*_*avg*_ and 30% when considering *V*_*min*_), whose equations are  given by:

6 $$ \begin{aligned}   V_{{avg}}  &  =  -  109  + 0.0 7 ET0 + 0.2 5 P \\    V_{{min}}  &  =  - 13 - 0.0 2 ET0 + 0.1 3 P \\    V_{{max}}  &  =  -  251 + 0.2 3 ET0 + 0.4 0 P \\  \end{aligned}  $$

## Results and discussion

### Calculation of the indicators

**Figure 1:**
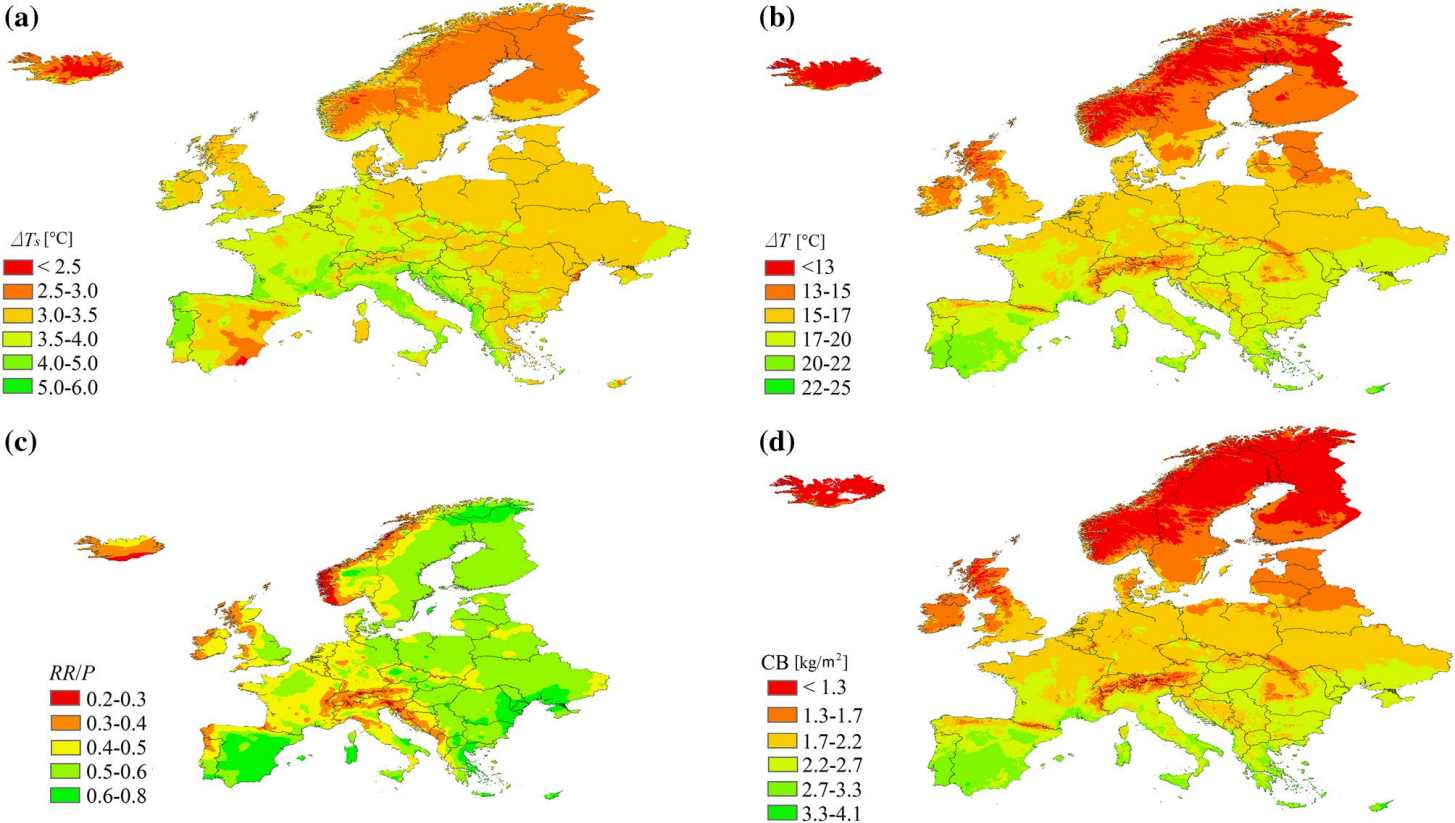
Maps of benefits per m^2^ across Europe for Δ*T*_*s*_ (**a**), Δ*T* (**b**), *RR/P* (**c**) and *CB* (**d**), in the present scenario.

**Figure 2:**
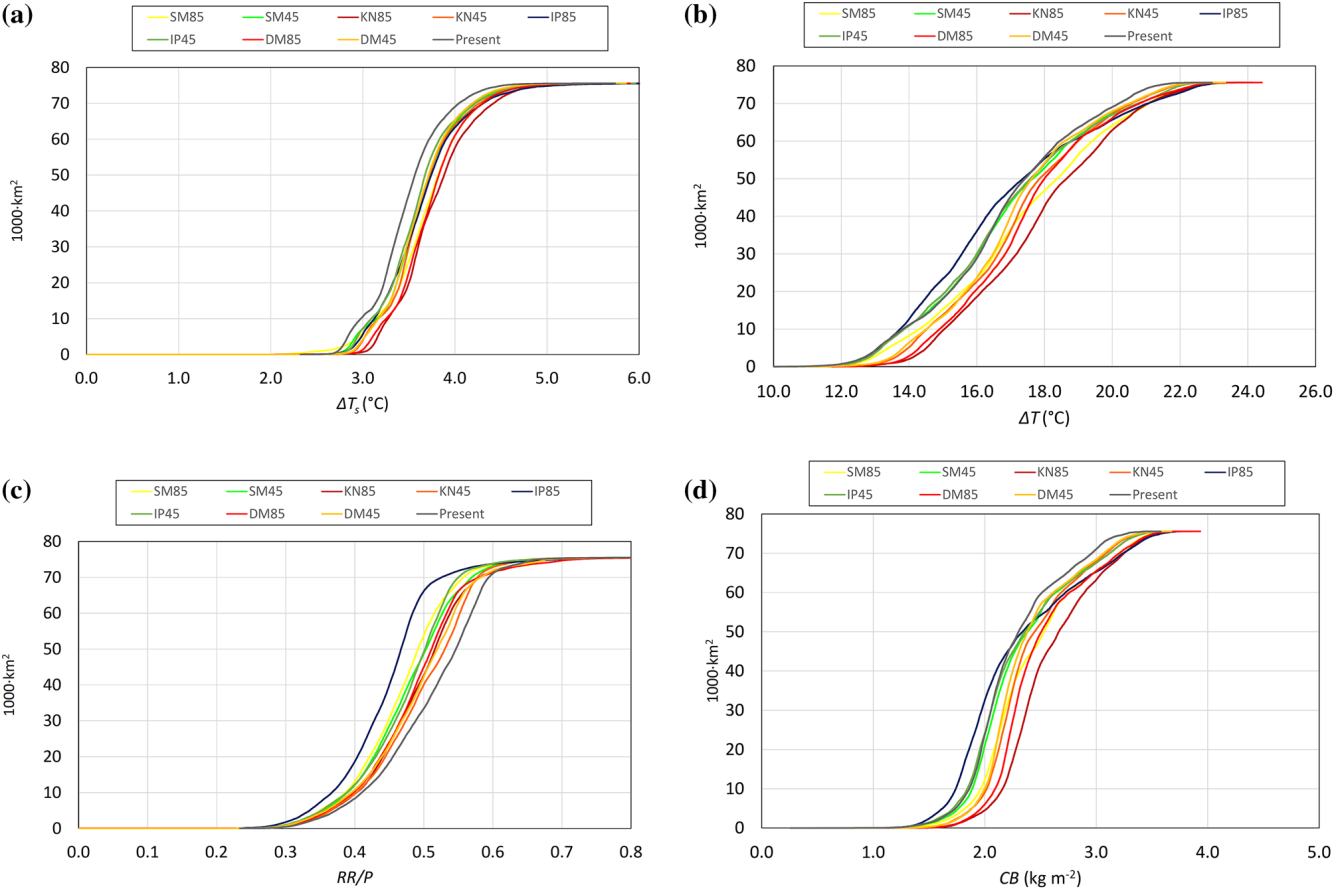
Cumulative curves of urban surfaces versus the indicator *ΔTs* (a), Δ*T* (b), *RR*/*P* (c) and *CB* (d). The black line represents present conditions, while lines in color stand each for one climatic scenario. The y-axis is the cumulative surface area of the present European urban areas.

Figure 1 shows the distribution of the urban greening benefit indicators computed at European scale under the current scenario, while Fig. 2 shows the cumulative distribution of impervious urban areas by increasing value of each indicator, under the current and future scenarios. It should be stressed that, while the indicators of Eqs. (1–4) are computed for every grid cell, the curves of Fig. 2 reflect also the spatial distribution of impervious urban surfaces, and hence they give more prominence to the values of the indicators in the most densely urbanized areas of the continent.

The reduction of surface temperature Δ*T*_*s*_ (Fig. 1a) is highest in the warmer and not excessively dry climates of Central and Southern Europe, reflecting the patterns of actual evapotranspiration. Most European urban areas would achieve temperature reductions of about 3–3.5 °C (Fig. 2a), slightly increasing with the severity of climate heating under the various scenarios, causing a reduction of sensible heat to the atmosphere, a driver of urban heat island effects, between 20 and 40% (see Appendix 1, Supplementary Material for further details). The highest temperature reduction at the roof surface, Δ*T,* is mostly perceived in the South of Europe (Fig. 1b), consistent with the pattern of potential evapotranspiration, similarly to the production of dry biomass *CB* (Fig. 1d). The reduction of temperature at the roof is predicted between 15 and 17 °C for most of Europe under the current scenario, and may increase of about 2 °C under the most severe climate scenario (Fig. 2b). Runoff reduction is significantly higher in areas with moderate precipitation, particularly in the plains, compared to rainier areas such as the Atlantic edge of the continent and high mountain ranges (Fig. 1c).

**Figure 3:**
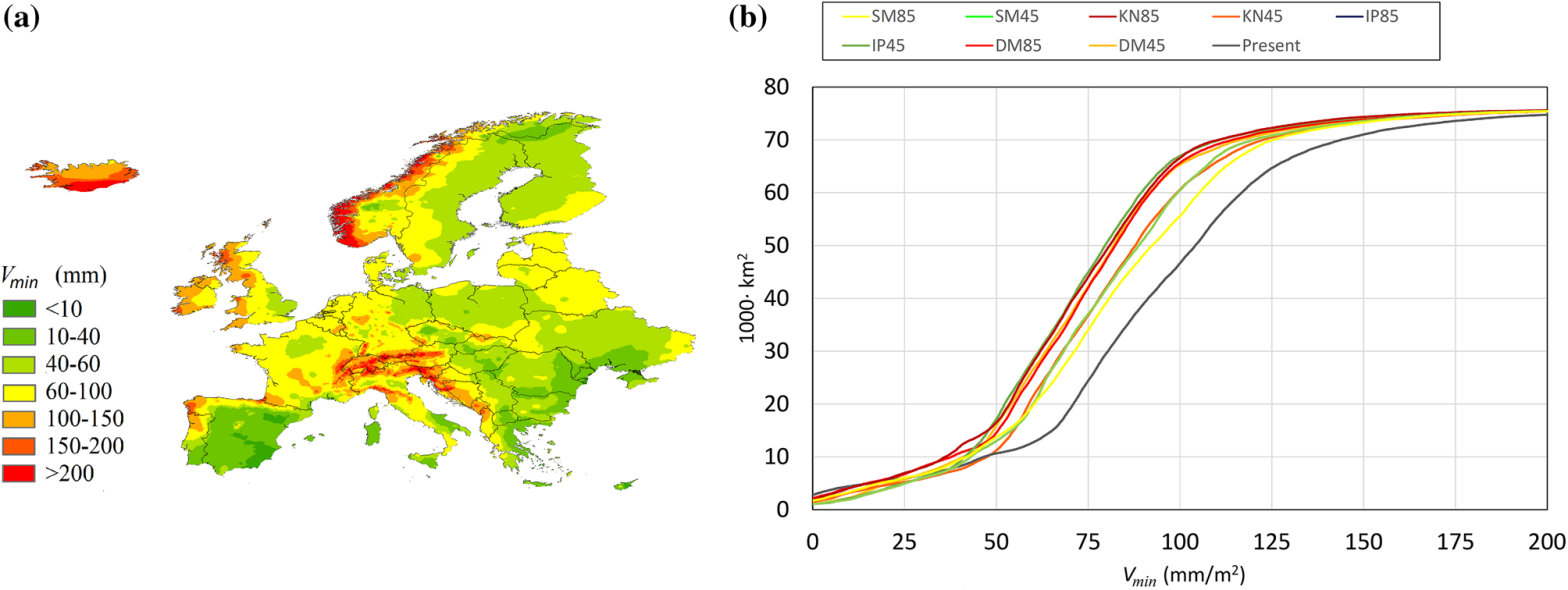
Storage volume *V*_*min*_ required to store the runoff in the case of constant demand.

**Figure 4:**
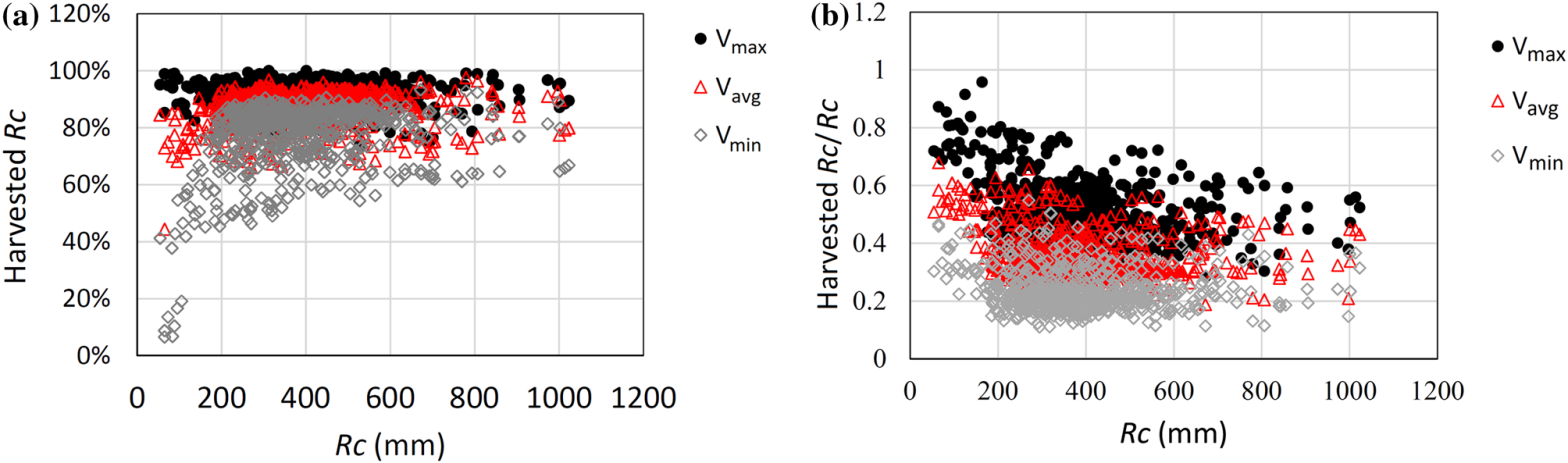
Runoff that could be harvested, and normalized storage volume *V*_*min*_ versus the annual average runoff (Rc) for the case of constant withdrawal, calculated throughout the 14 year time series.

The maximum storage volume, *V*_*max*_ , calculated by Eq. 6, would allow to reuse 92% of the annual runoff, while *V*_*min*_ and *V*_*avg*_ would allow to store 77% and 86% of the runoff, respectively, as resulting from a daily balance of the storage volume calculated over the 14 year time series. As the storage volume normalized to the annual runoff *R*_c_ is 0.24, 0.36 and 0.51 for *V*_min_, *V*_avg_ and *V*_max_, respectively (Figure 4b), choosing a storage volume equal to *V*_min_ appears to be the most cost-effective solution. *V*_*min*_ is mapped as shown in Fig. 3a for the case of constant demand, under the current scenario, while in Fig. 3b the volumes are plotted versus the cumulated areas.

### Physical and environmental implications

These potential effects of green surfaces at European scale correspond to potential benefits. The total benefits extrapolated for the EU are summarized in Table 1. Results are referred to the impervious surfaces corresponding to building roofs, that are assumed to amount to a total of 26,450 km^2^ as per Bódis et al.^40^ . This represents 35% of the European impervious surface. Although it is highly unlikely that the majority of the roofs may support a uniform soil cover of 30 cm, they could still bear patches of that thickness over a part of their surface. Moreover, additional surfaces such as sealed ground could be greened. Overall, having in mind these considerations, we pragmatically regard this 35% of impervious urban areas as a maximum extent that could be greened in Europe. All benefits calculated below would obviously scale proportionally for any reduction of the percentage of area subjected to greening. The quantification of Table 1 is explained below.

**Table 1:** Climatic descriptors and quantification of annual benefits at the European scale in the present and future climatic scenarios, assuming to green all roof surfaces, or 35% of the European impervious surfaces.

	Units	85KN	85SM	85IP	85DM	45KN	45SM	45IP	45DM	Present
Annual precipitation	mm	715	772	836	719	708	753	757	721	685
Annual *ET0*	mm	859	795	748	817	771	735	732	751	695
Runoff reduction	km^3^	10.0	11.4	12.9	10.2	9.8	10.9	10.9	10.1	9.3
Savings on energy needed for the cooling of buildings	bill. Є	19.6	19.1	18.3	19.2	19.0	18.5	18.5	18.8	18.4
CO_2_ emissions reduction (through energy saving)	Mtons	31.9	31.0	29.7	31.2	30.9	30.1	30.1	30.6	29.9
CO_2_ sequestration (through biomass)	Mtons	25.9	25.9	25.9	25.9	25.9	25.9	25.9	25.9	25.9
Total CO_2_ emissions reduction	Mtons	57.8	57.0	55.7	57.1	56.8	56.0	56.0	56.5	55.8

The reduction of land surface temperatures, Δ*T*_*s*_, reduces the thermal irradiation and convective heat flux from urban surfaces (see Appendix 1 of Supplementary Material), which are the drivers of the heat island effect^44^. As a first order approximation, the reduction of air temperature at 2 m from the surfaces can be expected to be about a half of Δ*T*_*s*_^45^ as an average value in summer. The reduction of air temperature would generate economic benefits, like the life cycle extension of electronic material and cars, benefits in the health and transport sectors, reduction of social stress and morbidity, and reduction of damages to trees and animals^46–48^.

The reduction of the surface temperature Δ*T* potentially reduces the cooling demand in summer (Eq. 5) by 92 TWh year^−1^. This energy saving corresponds to 29.9 Mtons of CO_2_ for the present scenario, considering emissions of 0.325 kg CO_2_ equivalent kWh^−1^ for European electricity^39^. Our estimate is arguably an upper limit of cooling energy savings. In many cases, underroof spaces of buildings are not cooled and effectively work already as an insulation, hence the reduction in the heat transferred from the roofs to underlying inhabited spaces may be lower than we estimate.

The yearly produced biomass *CB* is a benefit in itself whenever the biomass may be used (e.g. crops from urban agriculture). However, more importantly, it may be appraised in terms of carbon and carbon dioxide sequestration. The carbon dioxide sequestered from the atmosphere through biomass growth is 25.9 Mtons year^−1^ in the present scenario. This must be summed to the reduction of carbon emission following the expected decrease in cooling energy use for a total of 55.8 Mtons, or about 1.2% of the 4500 Mtons CO_2_ produced in the EU every year^37^.

It should be stressed that carbon dioxide sequestration by the biomass in green roofs is effective only if residues are not significantly degraded. This may be achieved by removing the biomass periodically before it undergoes respiration and mineralization. One could alternatively employ woody plants with a higher carbon accumulation capacity instead of herbaceous vegetation. Although our calculations are referred to a herbaceous annual crop, the results in terms of dry biomass would not be radically different had we considered a tree or shrub crop, as the dry matter potentially produced per unit surface is relatively independent of the plant^49^. On the other hand, trees and shrubs may be expected to have higher evapotranspiration, thus enhancing the benefits quantified here for a herbaceous crop.

If greening is implemented on about 35% of the impervious urban areas, we expect a reduction of runoff in the order of 17.5% compared to the total. Considering that pollutant loads associated to runoff are estimated in the order of about 30 million population equivalents (PE) in terms of biochemical oxygen demand (BOD), about 18 million PE in terms of total nitrogen and about 6 million PE in terms of total phosphorus ^6,35^, this can be a sizable contribution to the treatment of pollution from European urban areas. Besides the reduction of runoff volume, greened surfaces may also help reduce the frequency of combined sewer overflows because they buffer runoff and release it more slowly than impervious surfaces. This effect is arguably more important for smaller storm events, and tends to disappear as events cause the saturation of green roof storage.

It should be stressed that the above analysis considers a soil thickness of 30 cm on greened surfaces. Using the meta-models proposed in^30^ for the thickness of 10 cm we obtain a ratio between the indicators for thickness of 10 and 30 cm ranging between 80–97% for the reduction of surface temperatures, 55–57% for roof temperatures, 47–57% for biomass, and 84–86% for runoff. Soil thickness affects in particular the roof temperature, due to the associated thermal insulation effect, and the biomass, because a thicker soil can store a larger amount of water and allows a higher evapotranspiration for vegetation growth, while not impeding root growth. A comparison of different climate scenarios sheds light on the sensitivity of our results to the input climatic predictors (*P* and *ET0*). From Table 1, it can be calculated that the range (difference between the maximum and minimum value) of precipitation and potential evapotranspiration, as a percentage of the average value, is 20.3%, and 21.4% respectively. The corresponding ranges are 7% of the average for the cooling reduction, 3.7% for the reduced carbon dioxide emission, and 34% for the runoff reduction. The curves in Fig. 2 visualize the relatively small sensitivity of results to the climatic scenario.

### Economic implications

Most of the benefits of green roofs are collective. Only a few (e.g. energy saving in summer, and gardening) have an apparent private nature. The costs of greening roofs, on the contrary, are primarily borne by the private owners^50^. It has been observed that, in the absence of specific incentives, green roof implementation can be economically convenient only for specific commercial and multifamily buildings^25^. Therefore, private investments should be encouraged through appropriate fiscal and funding policies if the objective is to facilitate a mainstream uptake of this solution. In this section, an indicative cost-benefit analysis is carried out in order to shed light on the possible financing needs at stake, and considering to green the impervious surfaces covered by roofs.

The two main benefits that can be easily monetized are the avoided cost of cooling in summer (based on energy prices) and the reduction of carbon dioxide emissions (based on greenhouse gas emissions market prices). By summing the results of Eq. (5) for all gridcells in Europe where the greened surface is assumed to be 35% of the impervious urban area in the gridcell, cooling savings can reach 18.4 billion Є each year for the current scenario. For comparison, the current expenditure for residential cooling in summer can be assumed to be 78 billion Є year^−1^, based on an electricity use of 391 TWh^51^. Therefore, the cooling energy saving is 23.5% (18.4 billion Є/78 billion Є), in agreement with the results of Manso et al.^15^ for the value of 15% estimated for the hot-summer Mediterranean climate.

At the present carbon market price of 22.5 Є tons^−1^ (Ruf and Mazzoni^43^), the annual benefit related to the estimated reduction of greenhouse gas emissions corresponds to about 1.26 billion Є. It should be stressed how this is apparently an upper limit of this benefit, because not all greened surfaces may correspond to roofs of cooled building volumes, and because the biomass is likely to undergo at least a partial mineralization if not timely removed from the green surfaces. 

The benefit associated to the reduction of the heat island effect can also be quantified to some extent on the basis of existing literature studies, although their estimation is very complex and would require ad hoc studies. For example, for the city of Phoenix, this benefit was quantified in 80 € for 1 °C decrease per working resident, considering costs of electronic devices, maintenance of cars and performance of cooling^47^. In another analysis for the Melbourne area, the annual cost was quantified in 18 € per inhabitant, including health, transport, social distress, electric grid faults and damages to animal and trees^48^. In Malaysia, the annual cost of hazes, related to the urban heat island, was quantified in 12 € per habitant in 1997, including cost of illness, productivity loss, flight cancellation, tourism reduction, decline in fish landings, fire-fighting, cloud seeding and masks^46^. Therefore, costs can vary significantly among different contexts. Assuming conservatively a yearly benefit of 20 € for each of the ca. 559.5 million European urban inhabitants living in urban areas (75% of the total^52^), the Net Present Value (NPV) of this benefit over 40 years would be 221 billion € using a discount rate of 4%.

The cost of greening the roofs or other impervious surfaces is more difficult to quantify as it depends on several design details and site-specific conditions. For example, in Finland the cost ranges between 70 and 80 Є m^−2^, in Germany between 13 and 41 Є m^−2^, while in Switzerland around 20 Є m^−2^^53^. Assuming an average unit cost of 50 Є m^−2^, the costs to turn 26,450 km^2^ of impervious urban areas in Europe into green surfaces amounts to 1323 billion Є. This corresponds to an annual cost (discount rate 4%, 40 years life) of 63 billion euro. This means a cost of 6.3 € m^−3^ of annual runoff saved (assuming an average annual runoff saving of 10 km^3^), which is reasonably in line with an estimate of 9.2 € m^−3^ for the U.S. context, where the annual runoff volume reduction was 12%^54^ compared to our estimate of 17.5%.

Assuming a lifespan of 40 years^55^ and a discount rate of 4%^50^, the NPV of the cost saving of summer cooling over 40 years (18.4 billion Є year^−1^ in Table 1), that is the main private benefit of a green roof installed in a private building, is 364 billion Є (using a discount rate of 4%). The benefits of CO_2_ reduction, monetized in an emission trading system, would lead to a NPV of 24.85 ≈ 25 billion Є over 40 years (55.8 Mtons year^−1^). The NPV of the heat island benefit over 40 years would be 221 billion €. Deducting the sum of these benefits (totalling 610 billion €) from the estimated investment of 1323 billion €, yields a net gap of 713 billion Є, corresponding to an annual cost of about 60 € for each of the 559.5 million European citizens living in urban areas. This estimated annual cost is apparently affected by the uncertainty on green roof costs: it could reduce to 4 Є/year per urban citizen if the cost of the green roof is 25 Є m^−2^, and 129 Є/year per urban citizen if the cost is 80 Є m^−2^. An annual cost of 60 Є/year per urban citizen may be in many cases compensated by the additional benefits not quantified here. For example, the average increase of property value (rental prices) was estimated to be 8%^15^. Other benefits can be associated e.g. to leisure and recreation, socialization, amenity of the urban environment, and the creation of habitat or ecological connections in urban areas, besides the abovementioned positive effects in terms of water pollution and floods. Table 2 summarizes the economic results.

**Table 2:** Summary of benefits and costs of urban greening considered in this study for the European context.

Costs/benefits	Annual cost/saving	NPV(billion Euro)	Notes
Cost of greening	61.2	1323	Costs vary by a factor 0.5–1.6
Benefits from energy saving	18.4	364	Benefits depend on assumed cooling of buildings beneath greened surfaces
Benefits from heat island mitigation	11.2	221	1) Reduced costs: electronic devices, maintenance of cars, electric grid faults and damages to animal and trees, reduced productivity loss and flight cancellation, fire-fighting, masks; 2) Better performance of cooling, benefits on health, transport, tourism Assumed benefit: 20 €/urban resident per year
Benefits of GHG emission reduction	1.26	25	Benefits depend on energy saving (cooling) and assuming no mineralization of biomass
**Outstanding benefits of greening**			
Pollution and flooding reduction	Not quantified		
Health benefits	Not quantified		Partially included in heat island reduction
Recreation and wellbeing	Not quantified		
Support to biodiversity	Not quantified		
Improvement of urban landscapes (including value of properties)	Not quantified		Property value can increase by 8%^15^

**Figure 5:**
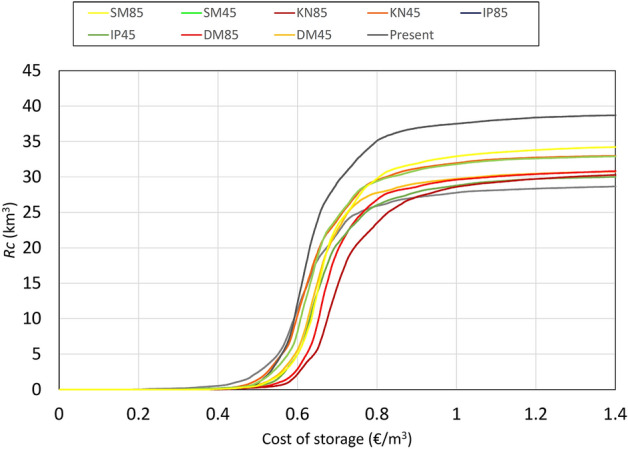
Cumulate runoff versus the cost of storage per unit of runoff, for a storage cost of 50 € m^−3^. Different climatic scenarios are shown.

The harvesting of runoff is a potential additional benefit, but it also entails costs. These can be quantified as a first approximation considering a cost of the storage volume *Cs* = 50 € m^−3^, a lifetime of the storage of 100 years, a discount rate of 4% and annual operation and maintenance costs of 3% of the investment. For a unit greened surface, the runoff potentially harvested equals *P*-*RR* and can be computed from Eq. 3, while the required storage volume to harvest it is given by Eq. 6. The cost of harvesting one m^3^ of runoff (marginal harvesting costs) follows from the abovementioned costing parameters. Figure 5 depicts the cumulate value of runoff as a function of the marginal harvesting cost. It can be seen that about 75% of the runoff can be harvested with marginal costs below 0.7 € m^−3^, a value compatible with urban water prices usually applied in Europe. *Cs* may be lower than 50 € m^−3^ , but often it may also be higher. Hence our calculation can be only regarded as a first indication and is accurate not more than within one order of magnitude. The quality of water from green surface runoff harvesting is arguably adequate for non-potable domestic use, but depends on the type of green roof and vegetation^13^.

## Conclusions

In this study, meta-models were used to estimate the maximum achievable benefits of urban greening at the European scale, focusing on converting 26450 km^2^, or 35% of the European urban surfaces into green surfaces

Our results show how green roofs may deliver significant benefits to European cities. They cool surfaces by between 2.5° and 6°, causing a reduction of sensible heat to the atmosphere, a driver of urban heat island effects, reducing air temperature of about 50% with respect to the surface temperature reduction. We estimate the benefits associated to surface temperature reduction at a NPV of 221 billion over 40 years. The reduction of heat flow to buildings corresponds to a potential cooling energy saving of about 92 TWh year^−1^ in the present scenario, which turns into energy cost savings whose upper limit is estimated at a NPV of 364 billion € over 40 years. The combined effect of carbon dioxide sequestration by biomass growing on green roofs, and energy savings can be up to 55.8 Mtons per year (present scenario), with a sequestration component of 25.9 Mtons year^−1^ (if biomass is removed or accumulated and not mineralized), yielding a NPV of avoided greenhouse gas emissions of about 25 billion € over 40 years . These monetized benefits, though, cover at best less than half of the costs of implementing urban greening, which we estimate to have a NPV of 1323 billion €. 

Urban greening has the potential to reduce urban runoff by about 17.5%, helping reduce urban diffuse pollution and the frequency of combined sewer overflows. As such, the role of green roofs should be considered in the context of river basin management. The residual runoff from green roofs could be in principle harvested and reused, but this would require an adequate storage capacity to buffer demand and availability. The costs of harvested runoff are usually expected to be below 0.7 € m^−3^, although the quality of water may not be sufficient for potable use.

We did not quantify the benefits related to runoff reduction and combined sewer overflow mitigation, nor to the economic value of the biomass beyond carbon sequestration, and our study does not explicitly address other benefits of urban greening, including possible increase of property values, socialization (e.g. related to community gardening) and wellbeing. Biodiversity improvement is also an important benefit, supporting pollination and improving the environmental quality of urban landscapes. Due to the fact that urban greening requires for a large part private investments, if we want to implement it on a large scale on European urban surfaces, we may need appropriate fiscal and funding policies. According to our quantification, the costs of greening not covered by the monetized benefits would be around 60 € per year per urban citizen. In many situations, the additional benefits not monetized in our study may be worth these costs. When this is the case, urban greening could represent a multifunctional no-regret, cost-effective solution meeting the aspirations of the European (and global) sustainability agenda.

## Supplementary Information

Supplementary Information 1.
