# Supplementary material for: Water, energy and climate benefits of urban greening throughout Europe under different climatic scenarios
Source: Sci Rep. 2021 Jun 9;11:12163. doi: 10.1038/s41598-021-88141-7 (PMC8190137; doi:10.1038/s41598-021-88141-7)
Supplement: Supplementary file 1 — Supplementary Information 1. [file 41598_2021_88141_MOESM1_ESM.docx]

Appendix 1

*Irradiant and convective heat flux estimation*

The reduction of irradiation power ∆*E* depends on the temperature of the surface before greening, *T_g_*, and after greening *T_g_* -Δ*T_s_*, according to the Stefan-Boltzmann equation, ∆*E*=σ[ε*T_g_*^4^-ε’(*T_g_* -Δ*T_s_* ) ^4^ ], with σ = 4.9 10^-9^ MJ m^-2^ K^-4^ d^-1^ (the Bolzmann constant) and emissivity values ε = 0.92 for grey surfaces and ε’ = 0.97 for green roofs. This is a highly non-linear function of the initial temperature *T_g_*. However, the relative change ∆*E* with respect to the irradiation power of a grey surface (*E_g_*) is a linear function of *AET* according to the following equation:

∆*E*/ *E_g_* = 0.000082 *AET* - 0.03 (A1)

In a similar way, the reduction of convective heat flux ∆*H* = *h*[*T_g_*-(*T_g_*- Δ*T_s_*)], where *h* is the convection coefficient, can be expressed as a function of potential evapotranspiration as:

∆*H /H_g_* = -0.000153 *ET0* + 0.42 (A2)

Equations A1 and A2 were obtained from data used Quaranta et al. (2021). For each FUA the temperature of the grey roof *T_g_*, averaged during summer months, and the temperature of a green roof (30 cm soil thickness and alfalfa) *T_g_-*∆*T_s_*, were computed. For each temperature, the irradiation was calculated using the Stefan-Boltzmann equation, obtaining ∆*E*. Finally, the term ∆*E*/*E_g_* was plotted versus the annual average actual evapotranspiration *AET*, to find the regression equation reported in Eq.A1. If the integration of the forth power of the hourly temperature would be used instead of the forth power of the average daily temperature, the discrepancy would be below 1% when the excursion of the daily temperature is below 10% of the daily average temperature (expressed in Kelvin). The irradiant heat flux ranges between -1.6% and 2.1%.

Equation A2 was derived in an analogous way, for each FUA. It is worth noting that ∆*H* depends on ∆*T_s_*, being ∆*H* = *h*∆*T_s_*, that, from Eq.1, depends on *AET*. Therefore, also ∆*H* is a function of *AET*. However, in Eq.A2 ∆*H* is normalized to *H_g_*, that depends on the roof temperature, that is a function of *ET0* rather than *AET* (Eq.2); therefore, ∆*H*/*H_g_* depends mostly on *ET0*, although ∆*H* is proportional to *AET*. The reduction of sensible heat to the atmosphere, a driver of urban heat island effects, ranges between 20% and 40%. The result on the reduction of sensible heat is in line with Alkama and Cescatti (2016), who found that the reduction of air mean temperature was about 70% (thus a 30% reduction of sensible heat) with respect to the mean temperature reduction at the land surface.
